# The Protective Effects of Pectic Polysaccharides on Dextran Sulfate Sodium-Induced Colitis in *Drosophila melanogaster* and Their Structure–Function Relationships

**DOI:** 10.3390/nu17101738

**Published:** 2025-05-20

**Authors:** Zhenou Sun, Tianyu Qi, Boyu Cheng, Yingxiao Guo, Dima Atehli, Steve W. Cui, Ji Kang, Qingbin Guo

**Affiliations:** 1 State Key Laboratory of Food Nutrition and Safety, College of Food Science and Technology, Tianjin University of Science and Technology, No. 29, 13th Avenue, TEDA, Tianjin 300457, China; zhenousun@tust.edu.cn (Z.S.); qty15109588841@163.com (T.Q.); chengboyu257@163.com (B.C.); gyx20220303@163.com (Y.G.); dimaatahla@163.com (D.A.); guoqingbin008322@tust.edu.cn (Q.G.); 2 Guelph Research and Development Centre, Agriculture and Agri-Food Canada, Guelph, ON N1G 5C9, Canada; steve.cui@agr.gc.ca

**Keywords:** pectic polysaccharide, ulcerative colitis, structure–activity relationship, molecular weight

## Abstract

Background: Pectic polysaccharides exhibit therapeutic potential against intestinal inflammation. However, the influence of structural variations on their efficacy remains largely unexplored. Methods: This study investigated the structural and anti-inflammatory relationships of okra pectin (OP), citrus pectin (CP), apple pectin (AP), and hawthorn pectin (HP). Based on FT-IR spectra, CP was identified as a high-methoxyl pectin, with a degree of methyl esterification (DM) of 72.07 ± 3.86%. OP, AP, and HP were low-methoxyl pectins with the following DM values: 19.34 ± 3.04%, 32.11 ± 1.71%, and 38.67 ± 2.75%, respectively. Results: Monosaccharide composition analysis revealed that OP exhibited the highest abundance of RG-I regions among all the samples. Homogalacturonan (HG) was the predominant structural region in AP and HP, while CP contained both of the aforementioned structural regions. Our findings demonstrated that OP and CP significantly ameliorated dextran sulfate sodium (DSS)-induced colitis in the wild-type *Drosophila melanogaster* strain w^1118^, as evidenced by improved intestinal morphology, reinforced intestinal barrier function, and enhanced locomotor and metabolic activity. These effects were mediated by the inhibition of JAK/STAT signaling and the activation of the Nrf2/Keap1 pathway. Notably, reducing the molecular weight of CP to 18.18 kDa significantly enhanced its therapeutic efficacy, whereas a reduction in OP molecular weight to 119.12 kDa extended its median lifespan. Conclusions: These findings first suggest that abundant RG-I structures and low molecular weight endowed pectins with significant anti-inflammatory activity.

## 1. Introduction

Inflammatory bowel disease (IBD) encompasses chronic diseases of the gastrointestinal system, primarily Crohn’s disease (CD) and ulcerative colitis (UC) [1]. UC is characterized by inflammation and ulcers in the inner layers of the colon and rectum [2]. Studies have indicated that multiple signaling pathways play a regulatory role in UC-associated immune responses and oxidative stress, including the Nrf2/Keap1 pathway [3], JAK/STAT pathway, and Toll signaling pathway [4]. The dextran sulfate sodium (DSS)-induced colitis *Drosophila melanogaster* (wild-type strain w^1118^) model serves as a valuable system for studying the mechanisms of UC and potential treatment strategies [4]. This is because the digestive system of fruit flies, comprising the anterior intestine, anterior brain, midgut, and hindgut, exhibits intestinal functions similar to those of mammals [5]. Moreover, it has been found that 75% of fruit fly genes have human homologs [6]. Consequently, fruit flies have become a powerful model for investigating intestinal physiology and pathology. Numerous products have demonstrated protective effects against sodium dodecyl sulfate and DSS-induced intestinal injury in flies, such as *Flos puerariae* [7] and *Allomyrina dichotoma* [8].

Pectic polysaccharides (PPs) are generally located between the primary cell walls and within the cell walls of higher plants [9]. PPs are complex molecules mainly composed of two structural regions: homogalacturonan (HG) and rhamnogalacturonan I (RG-I). HG is a linear homopolymer consisting of α-1,4-linked D-galacturonic acid (GalA) that can be acetylated and partially methyl-esterified [9]. The RG-I domain consists of a repeating disaccharide unit [→2)-α-L-Rha*p*-(1→4)-α-D-Gal*p*A-(1→]_n_ and side chains containing galactan, arabinan, arabinogalactan-I (AG-I), and arabinogalactan-II (AG-II) [9]. Dietary polysaccharides, such as PPs, exhibit protective effects against IBD. For example, pectin-type polysaccharides isolated from crabapples reduced the production and mRNA expression of pro-inflammatory factors in intestinal epithelial cells and improved symptoms of colitis in mice with DSS-induced colitis [10]. It has been demonstrated that pectin polysaccharides with different chemical compositions can attenuate inflammatory parameters [11]. Moreover, PPs are more effective in alleviating inflammation compared to other plant polysaccharides such as inulin and β-glucan [12]. However, the structural complexity of pectins has hindered in-depth investigation of their structure–activity relationships with respect to anti-inflammatory activity. Despite existing studies showing that PPs with more neutral side chains more effectively ameliorate colitis and related disorders in mice [13] and Carlos Sabater et al.’s demonstration that galactose content is particularly relevant for preserving pectin’s anti-inflammatory properties [14], the underlying structure–activity mechanisms have not been fully elucidated due to the intricate nature of pectin structures.

A comprehensive review of the literature indicates that the most studies on PPs have focused on basic structural characterizations, with limited investigation into their higher-order structures. Furthermore, structural modifications have rarely been applied to PPs with clearly defined anti-inflammatory effects. In this study, four PPs with specific structural characteristics were selected to investigate their structure–function relationships. We screened out two high-molecular-weight PPs that were effective in treating UC to further study the impact of molecular weight on the treatment. For this purpose, a DSS-induced colitis fruit fly model was used to determine changes in lifespan, locomotion, metabolism abilities, intestinal morphology, and related signaling pathways, and the anti-inflammation activity of the PPs was demonstrated.

## 2. Materials and Methods

### 2.1. Materials

Okra, citrus, apple, and hawthorn were purchased from Handan, Hebei Province, China. DSS was purchased from MP Biomedicals Co., Ltd. (Irvine, CA, USA). Galacturonic acid (GalA), rhamnose (Rha), galactose (Gal), arabinose (Ara), xylose (Xyl), mannose (Man), and glucose (Glc) were supplied by Sigma-Aldrich Co., Ltd. (Shanghai, China). The wild-type *Drosophila melanogaster* strain W^1118^ used in the experiment was purchased from the Fangjing *Drosophila* Center of the East China Institute of Peking University. All chemicals and solvents used in the study were of analytical grade.

### 2.2. Extraction and Preparation of PPs from Different Sources

Okra, citrus peel, apple, and hawthorn were processed (diced/dried/ground), defatted via triple ethanol extraction, acid hydrolyzed (pH = 2, 100 °C, 1:20 *w*/*v*), and centrifuged at 4000× *g* for 20 min at 25 °C. Proteins were removed by Sevag reagent, followed by ethanol precipitation (60%, 4 °C), dialysis (3500 Da), and lyophilization to obtain OP, CP, AP, and HP.

### 2.3. Structural Modification of PPs

We screened two high-molecular-weight PPs (CP and OP) that were effective in treating UC to further study the impact of molecular weight on treatment.

#### 2.3.1. Enzymatic Hydrolysis to Obtain Citrus Pectins with Different Molecular Weights

Citrus pectic polysaccharides (CPs) with different molecular weights were prepared by pectinase hydrolysis [15]. The enzymatic hydrolysis conditions were as follows: pectin concentration of 10.0 mg/mL, pectinase dosage of 0.05 mg/mL, temperature maintained at 50 °C, and treatment duration of 10 min or 30 min. The resulting hydrolysates were freeze-dried to obtain enzyme-hydrolyzed citrus pectin samples designated as CP1 (10 min hydrolysis) and CP2 (30 min hydrolysis).

#### 2.3.2. Ultrasound Treatment of Okra Pectic Polysaccharides to Obtain Different Molecular Weights

Okra PPs (OPs) with different molecular weights were prepared by a ultrasonic method [16]. The OP was treated with an ultrasonic cell disruptor (frequency: 25 kHz, maximum output power: 650 W). The concentration of OP was maintained at 5 mg/mL throughout the process. The ultrasonic parameters were as follows: pulse cycle of 3 s on/3 s off, ultrasonic power at 60% of maximum capacity (equivalent to 390 W), with total treatment durations of 5 min and 240 min, respectively. The resulting suspensions were freeze-dried to obtain ultrasonicated okra pectin samples, designated as OP1 (5 min treatment) and OP2 (240 min treatment).

### 2.4. Structural Characterization of PPs

#### 2.4.1. Chemical Composition Analysis

The total sugar content of the PPs was determined according to the phenol–sulfuric acid method using glucose as the standard [17]. Total uronic acid content was determined by the m-hydroxydiphenyl colorimetric method [18]. The soluble protein content was determined using a BSA protein assay kit [19]. The content of total phenolic compounds was determined using the modified Folin–Ciocalteu method with ferulic acid as a standard [20].

#### 2.4.2. Relative Molecular Weight Measurement

High-performance size exclusion chromatography (HPSEC) (SHIMADZU, Co., Kyoto, Japan) with a refractive index (RI) detector and ultrahydrogel linear column (10 μm, 7.8 mm × 300 mm, Waters, Co., Framingham, MA, USA) was used to determine the relative molecular weight of the PPs [21]. The ultrahydrogel linear column was used with a column temperature of 40 °C and a mobile phase of NaNO_3_ at a flow rate of 0.6 mL/min. The calibration curve was constructed using T-series dextran standards (T-10, 40, 70, 500, 2000 kDa).

#### 2.4.3. FT-IR Spectra

The FT-IR spectra were recorded using an IS50 FT-IR spectrometer (Nicolet, Green Bay, WI, USA) under a wavenumber range of 4000–400 cm^−1^, with 32 scans at a resolution of 4 cm^−1^. Then, 1 mg samples and 150 mg KBr were mixed, ground into a fine powder, and pressed into a pellet for FT-IR measurement [22]. The degree of methyl esterification (DM) in PPs was determined using Equation (1).

(1) $$\mathrm{DM}=\frac{A_{1740}}{A_{1740}+A_{1630}}$$

where *A*_1740_ and *A*_1630_ are the areas from the bands at 1740 cm^−1^ and 1630 cm^−1^, respectively.

#### 2.4.4. Monosaccharide Composition Analysis

The monosaccharide composition was determined using a high-performance anion-exchange chromatography-pulsed amperometric detector (HPAEC-PAD) (Dionex ICS-5000, Dionex Corp., Sunnyvale, CA, USA) [23]. Briefly, a 1 mg sample was hydrolyzed with 1 mL of 2 M trifluoroacetic acid (TFA) at 121 °C for 2 h. After diluting the hydrolysates with ultrapure water, the solution was passed through a 0.22 μm aqueous filter membrane and an activated solid-phase extraction column. The monosaccharide composition was then determined by ion chromatography and analyzed under the same conditions. Data collection and analysis were conducted using Chromeleon 6.8 software (Dionex Corp., USA).

#### 2.4.5. Methylation Analysis

Methylation analysis of CP, CP1, CP2, OP, OP1, and OP2 was conducted following the protocol outlined by Ciucanu [24]. The methylated polysaccharide was dried, hydrolyzed, reduced with sodium boron deuteride, and acetylated with acetic anhydride to yield partially methylated alditol acetates (PMAAs). Following filtration through 0.22 μm organic membranes, the derivatives were identified using a GC-MS system equipped with an HP-5 column (Agilent 7890 N, Agilent, Santa Clara, CA, USA).

### 2.5. DSS-Induced Colitis in Drosophila melanogaster

#### 2.5.1. Drosophila Strains and Rearing

Ethical approval was not required for the use of *Drosophila melanogaster* in this study. The experiments utilized the *Drosophila melanogaster* wild-type strain w^1118^. The emerged flies were reared on a basal medium composed of 20.4 g of corn flour, 15.6 g of sucrose, 1.68 g of yeast powder, 1.8 g of agar powder, 1.2 mL of propionic acid, and 190 mL of distilled water. They were maintained at 25 ± 1 °C with a relative humidity of 55 ± 5% under a 12 h light/12 h dark cycle [25,26].

#### 2.5.2. UC Model

The anti-UC efficacy of PPs was assessed using a *Drosophila* UC model [2]. Flies were randomly assigned to the following experimental groups: a control group, a DSS-treated group, and DSS-treated groups supplemented with PPs. Prior to the experiment, each group of flies was starved for 2 h before being transferred to new vials containing filter paper saturated with the respective media. The dietary composition for each group was as follows:

Control group: filter paper containing 5% (*w*/*v*) sucrose.
DSS group: filter paper containing 5% (*w*/*v*) sucrose and 5% (*w*/*v*) DSS.
PP-treated groups: filter paper containing 5% (*w*/*v*) sucrose, 5% (*w*/*v*) DSS, and PPs (0.5, 1, and 2 mg/mL).

#### 2.5.3. Survival Rate Assay

The 2-day-old female flies from the same generation (20 flies in each tube, 10 tubes in each group, 200 flies in each group) were divided into different groups (Control, DSS, and PP-treated groups). The number of dead flies was recorded daily, and the medium was replaced with fresh filter paper every 12 h until all flies had died [27]. The survival rate of flies in each group was recorded and calculated using the following formula.

(2) $$\mathrm{Survival}\;\mathrm{rate}\;(\%)=\frac{Number\;of\;surviving\;flies\;per\;time\;point}{Total\;number\;of\;flies\;at\;the\;beginning\;of\;the\;experiment}$$

#### 2.5.4. Climbing Assay

The climbing assay was performed according to a previously described method with minor modifications [1]. Briefly, 2-day-old female flies in the control group were treated with 5% (*w*/*v*) sucrose solution, the DSS group was treated with 5% (*w*/*v*) sucrose solution and 5% (*w*/*v*) DSS solution, and the PP-treated groups were treated with a 5% (*w*/*v*) sucrose and 5% (*w*/*v*) DSS solution containing PPs at 2 mg/mL for 48 h; the flies were transferred to empty plastic tubes (10 cm length × 2 cm diameter) and allowed to acclimatize for 5 min. The number of flies that climbed from the bottom of the tube to the top within 15 s was recorded. Each experimental group consisted of 5 vials containing 10 flies (50 flies in each group), with three biologically independent replicates.

#### 2.5.5. Food Consumption Assay

The UC model was established with the following groups: Control, DSS-treated, and PP-treated, maintained as per Section 2.5.4. Subsequently, adult flies were transferred to empty vials to starve for 12 h and then offered food containing 2% bromophenol blue for 4 h. The abdominal color of each fly was observed and scored under a light microscope as described in previous studies [5]. Each group consisted of 3 vials containing 10 flies each (n = 30 flies per group), with three biologically independent replicates.

#### 2.5.6. Measurement of Intestinal Length and Body Weight

The UC model was established with the following groups: Control, DSS-treated, and PP-treated, maintained as per Section 2.5.4. After 72 h of intervention, the filter paper was carefully removed. All flies were weighed, and the rate of weight change in each group was calculated using the following formula. Each experimental group consisted of 3 vials containing 20 flies each (n = 60 flies per group), with three biologically independent replicates.

(3) $$\mathrm{The}\;\mathrm{body}\;\mathrm{weight}\;\mathrm{change}=\frac{The\;body\;weight\;after\;treatment}{The\;body\;weight\;before\;treatment}$$

In addition, the intestines were dissected in paraformaldehyde, and their length was measured using a vernier caliper [4].

#### 2.5.7. Smurf Assay

A blue dye feeding experiment was utilized to assess the effects of PPs on the intestinal barrier of flies. Briefly, the UC model was established with the following groups: Control, DSS-treated, and PP-treated, maintained as per Section 2.5.4. After 72 h of intervention, the filter paper was carefully removed, followed by a 2 h fasting period. Subsequently, tissues were stained with brilliant blue dye solution for 12 h under standardized conditions [4]. A fly was classified as a “Smurf” when the dye coloration could be observed outside the digestive tract [5]. The leakage rate was calculated according to the percentage of blue fruit flies within the total fruit fly population. Each group consisted of three vials containing 10 flies (n = 30 flies per group), with three biologically independent replicates.

The blue dye stock solution was prepared by diluting edible blue dye to a concentration of 2.5% (*w*/*v*) using a 5% (*w*/*v*) sucrose solution and subsequently filtering it through a 0.22 μm filter membrane. The feeding solutions were prepared as follows:

Control: 2.5% erioglaucin disodium salt + 5% sucrose.
DSS: 2.5% erioglaucin disodium salt + 5% sucrose + 5% DSS.
PPs solution: 2.5% erioglaucin disodium salt + 5% sucrose + 5% DSS + 2 mg/mL PPs.

#### 2.5.8. Hematoxylin and Eosin (H&E) Staining

Bodily tissue specimens were taken from the *Drosophila*. The whole-body tissues were fixed using 4% paraformaldehyde, dehydrated, embedded, and sectioned. Paraffin sections were deparaffinized and rehydrated. Nuclei were stained with Harris hematoxylin (5 min), followed by washing, differentiation, bluing, and rinsing. Cytoplasm was counterstained with eosin. After dehydration and mounting, sections were examined microscopically [28].

#### 2.5.9. RT-qPCR Analysis

Total RNA was extracted from the intestines of each sample using an RNA extraction kit (Beijing Solarbio Science and Technology Co., Ltd., Beijing, China), and cDNA was then synthesized using a HiFiScript cDNA synthesis Kit (Jiangsu Kangwei Century Biotechenology Co., Ltd., Taizhou, China). RT-qPCR was performed using TB Green^®^ Ex Taq™II Premix Reagent (Tli RNaseH Plus) (Bao Biotechnology Dalian Co., Ltd., Dalian, China) on a real-time fluorescent quantitative PCR instrument (BIO RAD, Singapore). The 2^−ΔΔCt^ method was used with rp49 as the reference gene [29]. Primer sequences are listed in Supplementary Table S1.

### 2.6. Statistical Analysis

Data were analyzed using SPSS 26 software (IBM Corporation, Armonk, NY, USA) to determine the means and standard deviations. Graphs were generated using Origin 8.5 software (Microcal, Northampton, MA, USA), and group comparisons were analyzed by performing a one-way analysis of variance (ANOVA). A *p*-value < 0.05 was considered statistically significant.

## 3. Results

### 3.1. The Anti-Inflammatory Effects of PPs Were Screened in a Drosophila Model

#### 3.1.1. Structural Features of PP Samples

**Figure 1:**
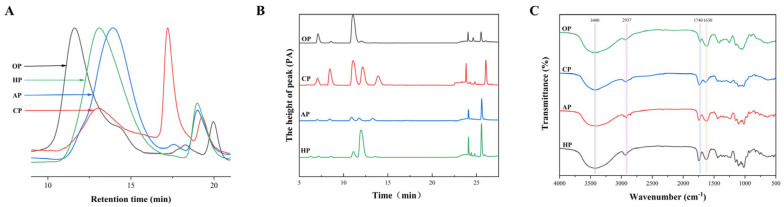
Structural features of four PPs: molecular weight distribution (**A**), monosaccharide composition (**B**), and FT-IR spectra (**C**).

The chemical properties of the four PPs are provided in Figure 1 and Table 1. All four types of PPs are acidic polysaccharides. OP had the highest molecular weight (Mw) among the tested samples at 4289.47 kDa. AP had the lowest Mw among the tested samples at 231.32 kDa. The FT-IR spectra of OP, CP, AP, HP exhibited typical pectin spectra with similar features (Table 1). The DM values for OP, CP, AP, and HP were determined by calculating the ratio of the peak area at 1740 cm^−1^ (COO-R) to the sum of peak areas at 1740 cm^−1^ and 1630 cm^−1^ (COO-) [22]. The determined values were 19.34 ± 3.04%, 72.07 ± 3.86%, 32.11 ± 1.71%, and 38.67 ± 2.75%, respectively. CP was a high-methoxyl pectin (HMP), while the other crude pectins (OP, AP, HP) were low-methoxyl pectins (LMPs).

As listed in Table 1, the content of GalA corresponds to the HG region. Gal, Rha, Glc, Ara, and Xyl form the side chains of the RG-I region [11]. According to Denman and Morris [30], the ratio of GalA/(Rha + Ara + Gal) (Ratio 1) represents the degree of linearity of pectin. The ratio of Rha/GalA (Ratio 2) represents the proportion of RG-I to pectin. Finally, the ratio of (Ara + Gal)/Rha (Ratio 3) reflects the degree of branching of RG-I. The Ratio 1 and Ratio 3 values for OP were the lowest, and the Ratio 2 value was higher than those for the other PPs. This indicates that OP was mainly composed of the RG-I domain, with a low degree of branching within this domain. OP contained 89.81% of the RG-I domain and 45.01 ± 5.79% galactose, which is primarily found in RG-I PPs as side chains. CP is a multicomponent heteropolysaccharide complex comprising 33.08% HG domains, 46.92% RG-I domains, and monosaccharide constituents of 12.91 ± 1.57% arabinose and 19.90 ± 2.71% galactose. It exhibits the highest Ratio 3 value (4.65) among tested polysaccharides, indicative of its hyperbranched architecture and pronounced structural complexity. AP and HP exhibited higher Ratio 1 values and lower Ratio 2 values, indicating that linear homogalacturonan (HG) occupies the main domain in these samples. Among them, AP contained 73.85% HG domain, and HP contained 58.33% HG domain. Some pectin fractions contained a certain amount of glucose. Hawthorn, in particular, has been demonstrated to contain significant quantities of other soluble sugars or non-pectin polysaccharides, which may be attributed to the presence of hemicellulose or starch in its peel or pulp [31]. Most of these domains are protective against dietary diseases. For example, pumpkin PP, which contains a linear α-1,4-D-galacturonic acid backbone, RG-I domain, and HG structure, significantly inhibited DSS-induced pathological changes by downregulating the TLR4/NF-κB and MAPK pathways [32]. In addition, it has been demonstrated that arabinose and galactose contents may determine the biological activity of pectin. Furthermore, ginseng polysaccharides with high GalA, galactose, and arabinose contents inhibit the expression of pro-inflammatory cytokines [33].

#### 3.1.2. PP Supplementation Increased Survival Rates

**Figure 2:**
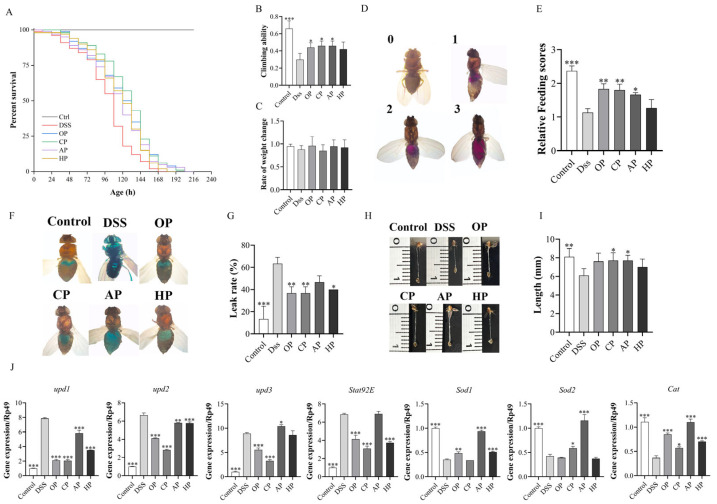
Effect of four PPs on DSS-induced inflammation in Drosophila. Survival rate curves (**A**), climbing ability (**B**), body weight (**C**), feeding score (**D**,**E**) (zero points: no blue dye in the abdomen; one point: light blue dye in the abdomen, less than one-third of the volume of the abdomen; two points: blue dye in the abdomen, one-half of the volume of the abdomen; three points: more than one-half of the volume of blue dye in the abdomen), leak rate (**F**,**G**), intestinal length (**H**,**I**), expression of JAK/STAT or Nrf2/Keap1 pathway-related genes in intestinal tissue (**J**). The *p*-value represents the significance of the differences between each group and the DSS-treated group. * *p* < 0.05, ** *p* < 0.01, *** *p* < 0.001.

To investigate the anti-inflammatory effect of PPs, the survival rates of fruit flies under DSS stimulation were determined. Dietary supplementation with PPs at concentrations of 0.5, 1, and 2 mg/mL significantly increased the lifespan in a dose-dependent manner (Table S2). When comparing the different pectins at 2 mg/mL, the CP group exhibited the highest average lifespan of 137.28 ± 6.20 h, while the OP group showed a higher average lifespan compared to the AP and HP groups (Figure 2 and Table S2). Notably, the CP group also exhibited the greatest median lifespan of 141.60 ± 13.15 h. In addition, all four pectin-treated groups (OP, CP, AP, HP) demonstrated higher survival rates compared to the DSS group, with the CP group exhibiting the highest survival rate among all interventions. This outcome aligns with prior research findings [27].

These findings suggest that the four PPs exert varying degrees of anti-inflammatory effects on DSS-induced flies. To further explore and compare the effects of different PPs, a consistent administration concentration of 2 mg/mL was used for all PPs in the subsequent index determination.

#### 3.1.3. PP Supplementation Improved Locomotion and Metabolism Abilities

To further determine the anti-inflammatory effect of different PPs on flies, the climbing ability, body weight, and food intake were measured in flies under DSS stimulation. The results showed that DSS treatment significantly reduced the climbing ability, with the climbing index of the DSS group decreasing by 0.36 compared to that of the control (*p* < 0.001). However, supplementation with 2 mg/mL of PPs could restore the reduced motility in *Drosophila* stimulated by DSS (Figure 2B). Compared with the DSS group, the climbing index of the OP, CP, and AP groups increased by 0.14, 0.16, and 0.16, respectively. Body weight and food consumption in flies can reflect their metabolic ability. Under DSS stimulation (Figure 2C), none of the pectin treatments showed a significant effect on the body weight of fruit flies. (*p* > 0.05). It was found that, with the exception of the HP group, the other PPs significantly increased the food intake of fruit flies stimulated by DSS (Figure 2C–E).

Therefore, the results indicated that OP, CP, and AP could rescue locomotor depression and metabolic disorder, while HP did not show a significant recovery effect (*p* > 0.05).

#### 3.1.4. PP Supplementation Protected Intestinal Morphological Integrity

Research indicates that the intake of DSS can induce inflammatory factors that damage the intestinal cells of fruit flies, leading to intestinal cell necrosis, a loss of barrier function, and a significant reduction in intestinal length [34]. The loss of intestinal barrier function in *Drosophila* can be detected using the non-absorbable blue food dye brilliant blue, which penetrates the intestinal epithelium and stains the entire body of *Drosophila* when intestinal permeability increases [34]. The results showed that DSS induced a significant increase in the proportion of “Smurf” flies, and the intestinal leakage rate in the DSS group increased by 50.00% (*p* < 0.001) compared with that in the control group, indicating that DSS compromised intestinal permeability (Figure 2F,G). Supplementation with OP, CP, and HP could significantly reduce the “Smurf” ratio. Compared to that in the DSS group, the intestinal leakage rates in the OP, CP, and HP groups decreased by 26.67%, 26.67%, and 23.33%, respectively. In contrast, the intestinal leakage rate in the AP group was reduced by 16.67%, which was not statistically significant (*p* > 0.05).

As showed in Figure 2H,I, the intestinal lengths measured across experimental groups were as follows: Control group (8.10 ± 0.89 mm), DSS group (6.10 ± 0.74 mm), OP group (7.60 ± 0.89 mm), CP group (7.70 ± 0.84 mm), AP group (7.70 ± 0.57 mm), and HP group (7.00 ± 0.87 mm). The intestinal length in the control group exhibited normal morphology. In contrast, the DSS group showed a significant reduction in intestinal length (*p* < 0.01), with a shortening of 24.69% compared to that in the control group. Compared to the DSS group, the intestinal lengths of fruit flies fed with different pectins were increased. Specifically, the OP, CP, AP, and HP groups showed increases of 24.59%, 26.23%, 26.23%, and 14.75%, respectively.

**Figure 3:**
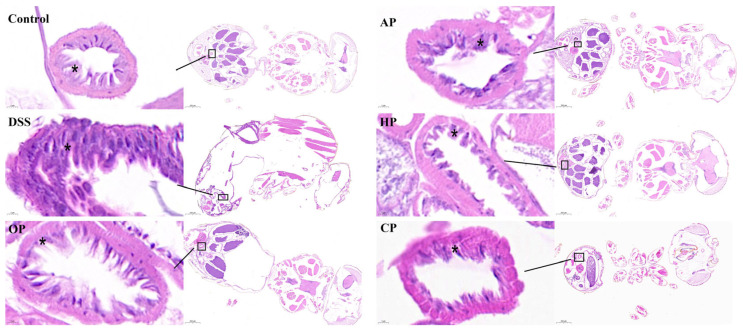
Histopathological examination (HE) of the *Drosophila* gut (n = 5 for each group). Nuclei were stained with Harris hematoxylin (left: magnification ×1700, right: magnification ×60).

Histopathological examination (HE) of the *Drosophila* gut can reveal tissue injuries in the intestinal wall [28]. The results showed that the intestines of DSS-induced flies exhibited severe morphological damage, including crypt damage, mucosal erosion, ulceratin, and mononuclear cells (* area in Figure 3). In contrast, PP treatments remarkably mitigated the morphological alterations caused by DSS (Figure 3). OP and CP induced mucosal healing, characterized by the complete resolution of erosions and ulcerations. In contrast, AP and HP led to a state of clinical remission or a continued clinical response. The results indicate that all the PPs improved intestinal permeability under DSS stimulation. Among them, OP and CP significantly alleviated intestinal atrophy, enhanced intestinal morphology, and improved resistance to external stimuli.

#### 3.1.5. PP Supplementation Alleviates DSS-Induced Intestinal Damage by Regulating Related Signaling Pathways

This study further explored the molecular mechanisms by which pectin confers protection against intestinal damage [4,35]. The JAK/STAT signaling pathway was identified as a core pathway involved in the therapeutic effects of PPs on UC (Figure 2J). The JAK/STAT signaling pathway in *Drosophila* includes three cytokines, namely upd, upd2, the upd3; a cytokine receptor (Dome); a downstream JAK (Hop); and STAT [4]. Compared to the control group, the DSS stimulation group exhibited significantly increased expression of *upd1*, *upd2*, *upd3*, and *Stat92E* in the gut (Figure 2J). Supplementation with OP, CP, and HP significantly reduced the mRNA expression levels of these genes. However, the AP group showed no significant reduction in the mRNA expression levels of *upd3* and *Stat92E* (Figure 2J). This suggests that OP, CP, and HP function to protect intestinal homeostasis by inhibiting the JAK/STAT pathway.

Many studies have suggested a strong association between oxidative stress and inflammation. The Nrf2/Keap1 pathway acts as a protective pathway against oxidative damage by promoting the gene expression of many antioxidant enzymes, such as superoxide dismutase (sod) and catalase (cat) [3]. Compared to the control group fed 5% sucrose, DSS-treated flies had dramatically decreased mRNA levels of *Sod1*, *Sod2*, and *Cat* in the intestine (*p* < 0.001), while PP supplementation remarkably rescued the decreased expression levels of these genes (Figure 2J). In particular, supplementation with AP significantly increased the expression levels of these genes (Figure 2J). Therefore, these results suggest that PPs can alleviate DSS-induced intestinal oxidative damage by activating the Nrf2/Keap1 pathway.

These findings suggest that PPs alleviated DSS-induced intestinal injury via multiple pathways. OP, CP, and HP alleviated DSS-induced inflammation in fruit flies by modulating the JAK/STAT and Nrf2/Keap1 pathways. AP mainly alleviated inflammation in *Drosophila* stimulated by DSS by promoting the Nrf2/Keap1 pathway.

### 3.2. Structural Modifications of OP and CP Were Performed, and Their Anti-Inflammatory Effects Were Investigated in a Drosophila Model

#### 3.2.1. Structural Features of Different-Molecular-Weight PP Samples

**Figure 4:**
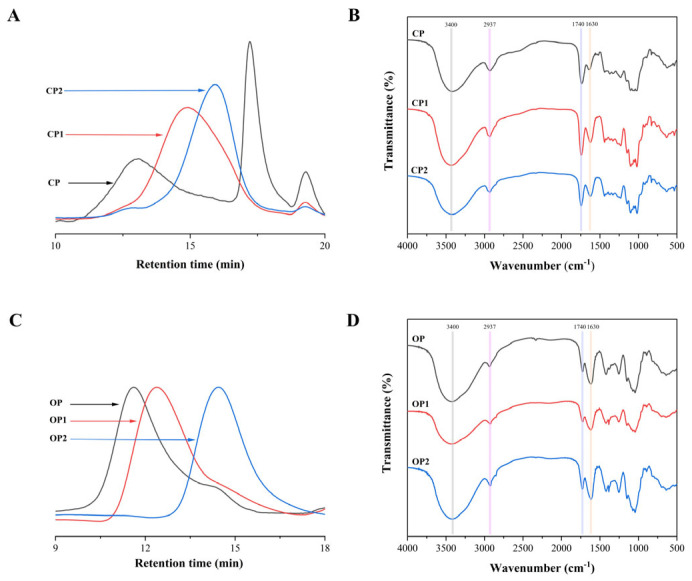
Structural features of different-molecular-weight PPs: molecular weight distribution (**A**,**C**) and FT-IR spectra (**B**,**D**).

Following enzymatic digestion, the relative molecular mass distribution of CP became more homogeneous, and the peak shape narrowed, yielding molecular weights of 65.68 kDa and 18.18 kDa, respectively (Figure 4).

While the monosaccharide types in pectin remained unchanged after enzymatic hydrolysis, their content exhibited significant or no alterations (Table 2). The FT-IR spectra of protopectin and enzymatically hydrolyzed pectin are presented in Figure 4. The peak area of modified pectin at 1630 cm^−1^ was significantly larger than that of the original pectin, and the change in peak area at 1740 cm^−1^ was smaller, indicating that the DM values of modified pectin were smaller than those of the original pectin [22]. During the enzymatic hydrolysis process, the backbone and side chains of pectin underwent varying degrees of cleavage, resulting in an increase in the relative content of galacturonic acid from 40.13 ± 6.51% to 60.77 ± 2.34% and 56.59 ± 0.49%. Concurrently, the DM of pectin was reduced, allowing for the release of previously bound galacturonic acid units [36]. Methylation analysis revealed that CP, CP1, and CP2 exhibited similar structures with multiple linkage patterns. The dominant linkage pattern in CP, CP1, and CP2 was →4)-Gal*p*A-(1→ (Table 3).

Ultrasonic treatment did not alter the types of characteristic functional groups, monosaccharide composition, or glycosidic bonds of polysaccharides. Moreover, it did not decrease the molecular weight or alter the chemical composition ratio or degree of polymerization [37]. After ultrasonication, the molecular weight of OP decreased from 4289.47 kDa to 1620.59 kDa and 119.12 kDa (Figure 4C). As shown in Figure 4D, there was no significant difference in the monosaccharide content or FT-IR spectra of okra pectin after ultrasound treatment. The methylation analysis revealed that predominant sugar residues in OP, OP1, and OP2 were →4)-Gal*p*A-(1→ (Table 3). Arabinose (α-Ara*f*) and/or galactose (β-Gal*p*) may be present at positions O-3 and/or O-4 in rhamnose. Based on the monosaccharide composition and residue analysis, it can be inferred that OP, OP1, and OP2 were pectins primarily composed of the RG-I domain [38].

#### 3.2.2. Effect of Different-Molecular-Weight PP Supplementation on Survival Rate

**Figure 5:**
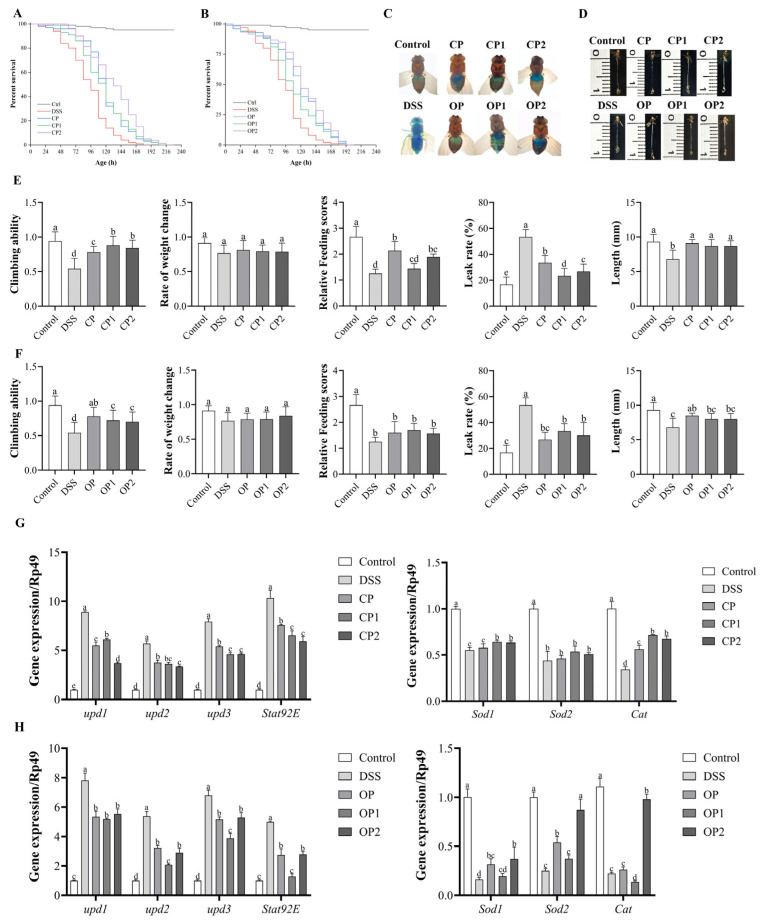
Effect of CP and its derivatives on survival rate curves (**A**), climbing ability, body weight, feeding score, “Smurf” ratio, and intestinal length (**C**–**E**) in DSS-induced *Drosophila*. Effect of OP and its derivatives on survival rate curves (**B**), climbing ability, body weight, feeding score, leak rate, and intestinal length (**C**,**D**,**F**) in DSS-induced *Drosophila*. Expression of JAK/STAT pathway-related genes (**G**) and Nrf2/Keap1 pathway-related genes (**H**) in intestines. Data with different letters exhibited significant differences (*p* < 0.05).

To investigate the anti-inflammatory effects of PPs with varying molecular weights, the survival rate and lifespan of female flies under DSS stimulation were assessed (Figure 5). These PPs, at a concentration of 2 mg/mL, enhanced the lifespan of female *Drosophila* (Table S3). As shown in Figure 5A, the average lifespan extension rates for CP, CP1, and CP2 were 24.14%, 21.40%, and 37.16%. Compared to the CP group, the CP1 group showed no significant changes in average lifespan (*p* > 0.05), and its survival rate also remained unchanged. The average lifespan extension rate in the CP2 group increased by 13.02% compared to that in the CP group. The survival rate of the CP2 group was higher than that of both CP and CP1.

Compared to OP-treated flies, OP1-treated flied exhibited a reduced lifespan extension rate, whereas the OP2 group showed an increased rate. Notably, the OP2 group’s median lifespan extension rate was 8.34% higher than that of the OP group. Figure 5B illustrates that the OP2 group had a higher survival rate than both the OP and OP1 groups.

These results indicated that reducing the molecular weight of CP to 18.18 kDa significantly extended the lifespan and survival rate of fruit flies. OP at 119.12 kDa had some effect on extending the median lifespan and survival rate of DSS-treated flies.

#### 3.2.3. Effect of Different-Molecular-Weight PP Supplementation on Locomotion and Metabolism Abilities

To further assess the anti-inflammatory effects of different-molecular-weight PPs in *Drosophila*, climbing ability, body weight, and food intake under DSS stimulation were measured. The results showed that DSS treatment remarkably decreased the climbing ability of adult flies, with the climbing ability index of the DSS group decreasing by 0.4 compared to that of the control group. However, supplementation with 2 mg/mL of PPs with different molecular weights could restore the reduced motility capacity in flies stimulated by DSS (Figure 5). Compared to that in the DSS group, the climbing ability indices of the CP, CP1, and CP2 groups increased by 0.24, 0.34, and 0.3, respectively. The indices of the CP1 and CP2 groups were higher than that of the CP group. Under DSS stimulation (Figure 5E), pectin treatment had no significant effect on the body weight changes of fruit flies (*p* > 0.05). It was also found that the CP, CP1, and CP2 treatments all increased the food consumption of fruit flies under DSS stimulation, although there was no significant difference between these groups (*p* > 0.05).

Compared with that of the DSS group, the climbing index of the OP, OP1, and OP2 groups increased by 0.24, 0.18, and 0.16 respectively. The climbing index of the OP1 and OP2 groups was lower than that of the OP group, but there was no significant difference between these groups (*p* > 0.05). Under DSS stimulation (Figure 5F), pectin treatment had no significant effect on the body weight changes of fruit flies (*p* > 0.05). The OP, OP1, and OP2 treatments increased the food consumption of flies under DSS stimulation, but there was no significant difference between these groups (*p* > 0.05).

Experiments showed that reducing the molecular weight of CP improves the exercise capacity of DSS-induced flies, but has little effect on metabolic capacity. Reducing the molecular weight of OP had little effect on the exercise capacity and metabolic capacity of the DSS-induced flies.

#### 3.2.4. Effect of Different-Molecular-Weight PP Supplementation on Intestinal Morphological Integrity

To investigate the protective effect of PPs with varying molecular weights on UC, the intestinal integrity of *Drosophila* was assessed using the brilliant blue assay, as shown in Figure 5E,F. The intestinal leakage rates of the control group and DSS-treated group were 16.67 ± 5.77% and 53.33 ± 5.77%, respectively. Compared to that of the control group, the intestinal leakage rate of the DSS-treated group increased by 36.66% (*p* < 0.001), indicating that DSS compromised intestinal permeability. Supplementation with CP, CP1, and CP2 significantly reduced the proportion of “Smurf” flies (Figure 5E), with corresponding intestinal leakage rates of 33.33 ± 5.77%%, 23.33 ± 5.77%, and 26.67 ± 5.77%, respectively. Notably, the intestinal leakage rates in *Drosophila* administered CP1 and CP2 were lower than that in the CP group.

The intestinal lengths of the control group, DSS group, CP group, CP1 group, and CP2 group were 9.30 ± 1.10 mm, 6.80 ± 1.30 mm, 9.10 ± 0.55 mm, 8.70 ± 0.97 mm, and 8.70 ± 0.76 mm, respectively. The control group exhibited longer intestinal length with normal morphology. In contrast, the DSS group showed a significant reduction in intestinal length, shortened by 36.76% compared to that of the control group (*p* < 0.01). Furthermore, compared to the DSS group, the intestinal length of *Drosophila* fed with CPs of different molecular weights increased by 27.94% in the CP1 group and 27.94% in the CP2 group.

Similarly, supplementation with OP, OP1, and OP2 significantly reduced the “Smurf” ratio (Figure 5F), and their respective intestinal leakage rates were 26.67 ± 5.77%, 33.33 ± 5.77%, and 30.00 ± 10.00%. Interestingly, the intestinal leakage rates of fruit flies administered OP1 and OP2 were higher than that of the OP group. The intestinal lengths of the OP group, OP1 group, and OP2 group were 8.50 ± 0.35 mm, 8.00 ± 0.79 mm, and 8.00 ± 0.79 mm, respectively. Compared with the DSS group, the intestinal length of fruit flies in the OP, OP1, and OP2 groups increased by 25.00%, 17.65%, and 17.65%, respectively. No significant differences were observed among these OP groups (*p* > 0.05).

These results indicate that the administration of low-molecular-weight citrus pectin (CP1 and CP2) was more effective in improving intestinal permeability. While applying OPs of different molecular weights could also improve intestinal permeability and maintain the intestinal morphology of the DSS-induced flies, there was minimal change observed upon reducing the molecular weight.

#### 3.2.5. Different-Molecular-Weight PP Supplementation Alleviates DSS-Induced Intestinal Damage by Regulating Related Signaling Pathways

The results demonstrated that within the JAK/STAT signaling pathway, DSS stimulation led to a notable upregulation of inflammation-associated genes, including *upd1*, *upd2*, *upd3*, and *Stat92E*, in the intestinal tissues (Figure 5G). By contrast, supplementation with CP, CP1, and CP2 resulted in a significant downregulation of the mRNA expression levels of these genes (Figure 5G). Specifically, compared to CP, CP1 and CP2 with reduced molecular weights not only significantly decreased the expression of *upd1*, *upd2*, *upd3*, and *Stat92E* but also upregulated the expression of *Sod1* and *Cat*. Moreover, OP1 significantly suppressed the expression of *upd2*, *upd3*, and *Stat92E*, while OP2 remarkably increased the expression of *Sod2* and *Cat* (Figure 5H). Previous studies have shown that PPs of different molecular weights can regulate inflammation by modulating related signaling pathways [16]. Our study confirmed that reducing the molecular weight of CP to 18.18 kDa enhances its ability to inhibit the JAK/STAT signaling pathway and promote the gene expression of the Nrf2/Keap1 signaling pathway. However, reducing the molecular weight of OP to 119.12 kDa primarily protected the intestine from damage by promoting gene expression in the Nrf2/Keap1 signaling pathway. Considering these findings, it is speculated that reducing the molecular weight of pectin can modulate its anti-inflammatory activity through the JAK/STAT and Nrf2/Keap1 signaling pathway.

## 4. Discussion

Our findings corroborate that the differential effects of PPs on DSS-induced intestinal inflammation are highly structure dependent [11]. The source and extraction method of pectin significantly influence its structural characteristics, including its domain ratio, monosaccharide composition, esterification degree, and molecular weight, thereby impacting its biological activity [39]. From a structural perspective, the main sugar residue of all four PPs is 4-galacturonic acid (4-Gal*p*A) [40], and they are all acidic polysaccharides. Compared to neutral polysaccharides, acidic polysaccharides generally exhibit superior anti-inflammatory activity [13]. The PPs exhibited differing quantities of HG and RG-I domains, as well as differences in the abundance of their respective side chains. These structural variations constitute the fundamental prerequisites for the manifestation of their distinct anti-inflammatory activities [39].

The administration of the four different PPs was capable of alleviating DSS-induced intestinal inflammation to varying extents. Notably, CP and OP effectively prolonged the lifespan of fruit flies, rescued locomotor depression and metabolic disorder, improved intestinal morphology, and protected the integrity of the intestinal barrier by inhibiting the JAK/STAT pathway and enhancing the Nrf2/Keap1 pathway. This protective effect may be attributed to CP and OP possessing homogeneous proportions of HG, RG-I, arabinose, and galactose [35], along with a highly branched structure, which has been associated with better protection against colitis [41]. CP is a multicomponent heteropolysaccharide complex comprising 33.08% HG domains, 46.92% RG-I domains, and monosaccharide constituents of 12.91 ± 1.57% arabinose and 19.90 ± 2.71% galactose, with a high degree of branching. The uniform and diverse domain structure, coupled with its high degree of branching, likely allows CP to confer the most pronounced protective efficacy against DSS-induced intestinal injury. OP contained 89.81% RG-I structures, and the monosaccharide ratio was GalA/Rha/Gal = 2.9:2.1:4.5. It has been reported that PPs with a high galactose content may exhibit enhanced biological activity in protecting the intestinal barrier from chemical damage [35], suggesting that the abundant galactose in OP may contribute to regulation intestinal barrier function. Furthermore, the RG-I structure of pectin plays a crucial role in in vitro fermentation for the production of short-chain fatty acids, which are important for maintaining intestinal health, modulating immune responses, inhibiting the growth of pathogenic bacteria, and promoting energy metabolism [42]. However, the function of PPs did not appear to be directly correlated with the total amount of RG-I, and OP had the lowest branching degree (Ratio 3 = 2.30) among the four pectins and contained almost no total phenols. These factors might explain why CP was more effective than OP in treating intestinal inflammation. In contrast, AP and HP, which were mainly composed of HG domains and contained only small amounts of arabinose and galactose (with HP also containing a large amount of other soluble sugars or non-pectin polysaccharides), demonstrated limited inhibitory effects on intestinal inflammation in fruit flies [31]. This compositional difference likely contributes to their lower anti-inflammatory activity.

It is well-established that the molecular weight of natural polysaccharides is closely related to their biological activity [16]. Upon reducing the molecular weight of CP and OP, our results indicated the following: While the molecular weight of CP decreased significantly from 676.57 kDa to 65.68 kDa, its efficacy in alleviating DSS-induced intestinal morphological damage did not significantly improve. However, when the molecular weight was further reduced to 18.18 kDa, the effects of alleviating the DSS-induced intestinal inflammation and promoting intestinal barrier function were significantly enhanced. These findings align with studies suggesting that lower Mw and DM, along with higher GalA content, can lead to a more effective protective role against IBD [40]. After enzymatic hydrolysis, the GalA content of CP1 and CP2 increased, and the DM decreased. Interestingly, the effect of CP1 in alleviating the DSS-induced intestinal morphological damage was not as pronounced as that of CP2, suggesting that the specific molecular weight range of CP may play a critical role in mitigating intestinal inflammation in flies. This observation is consistent with previous research indicating that that Angelica polysaccharides exhibit stronger anti-inflammatory activity upon molecular weight reduction [22] and that low-molecular-weight polysaccharides possess high anti-inflammatory activity [43]. Regarding OP, reducing its molecular weight from 4289.47 kDa to 1620.59 kDa did not significantly alter its effect on alleviating DSS-induced intestinal morphological damage. Polysaccharides with high molecular weights (molecular weight > 10^5^ Da) often exhibit relatively low solubility, hindering transmembrane absorption or affinity for immune cell carbohydrate receptors, thereby limiting their bioactivity [37]. Furthermore, their swelling and water-holding capacity can make them resistant to digestion and absorption, consequently reducing their bioavailability [37]. The anti-inflammatory effect of OP may be more closely related to its fine structure [42], such as its rich RG-I domains, high galactose content in monosaccharides, and lower DM. Notably, when the molecular weight of OP was reduced to 119.12 kDa, it significantly enhanced the Nrf2/Keap1 pathway and extended the median lifespan of *Drosophila*. One plausible explanation is that PPs require an appropriate molecular weight to effectively exert their optimal anti-inflammatory activity [16]. Additionally, studies have shown that the abundance of RG-I structures and low Mw of pectin can confer significant prebiotic activity [11,42], which may also contribute to the strong anti-inflammatory effect of low-molecular-weight okra pectin on DSS-induced flies.

## 5. Conclusions

Our study confirmed that the effects of PPs on a DSS-induced inflammation model are significantly influenced by their structural differences. Specifically, CP, characterized by a homogeneous proportion of HG, RG-I, arabinan, and galactan, along with a high degree of branching, demonstrated the most potent protective effects against DSS-induced intestinal injury. Furthermore, the molecular weight of CP played a crucial role in its anti-inflammatory efficacy. Low-molecular-weight CP2 exhibited a significantly enhanced ability to alleviate intestinal inflammation compared to the original CP sample by effectively modulating the JAK/STAT and Nrf2/Keap1 pathways. OP, which has almost 50% RG-1 structures, also demonstrated significant efficacy in treating UC. In conclusion, the abundance of RG-I structures and low Mw of PPs contributed to their enhanced effectiveness in mitigating UC. In contrast, PPs primarily composed of the HG domain, such as AP and HP, exhibited relatively limited efficacy in alleviating DSS-induced inflammation. This study elucidates the structural and anti-inflammation relationships, as well as the underlying pathways, of PPs derived from different sources with respect to intestinal damage. This provides a valuable dataset for considering the clinical application of PPs in the context of UC in human subjects.

## Figures and Tables

**Table 1:** Chemical composition of four pectin polysaccharides.

Chemical Features	OP	CP	AP	HP
Rha (%)	20.89 ± 0.71 ^a^	7.05 ± 1.67 ^b^	2.89 ± 0.05 ^c^	1.76 ± 0.14 ^c^
Ara (%)	3.03 ± 0.66 ^c^	12.91 ± 1.57 ^a^	4.91 ± 1.83 ^b^	1.12 ± 0.33 ^d^
Gal (%)	45.01 ± 5.79 ^a^	19.90 ± 2.71 ^b^	8.19 ± 1.55 ^c^	5.38 ± 0.28 ^c^
Glu (%)	2.42 ± 1.30 ^c^	13.25 ± 1.74 ^b^	4.53 ± 0.45 ^c^	30.93 ± 3.99 ^a^
Xyl (%)	-	6.76 ± 1.18 ^a^	2.73 ± 1.41 ^b^	0.72 ± 0.10 ^c^
GalA (%)	28.66 ± 4.63 ^d^	40.13 ± 6.51 ^c^	76.74 ± 1.47 ^a^	60.09 ± 4.64 ^b^
% HG = (GalA − Rha)	7.77	33.08	73.85	58.33
% RG-I = (2Rha + Ara + Gal)	89.81	46.92	18.89	10.01
Ratio 1	0.42	1.01	4.80	7.28
Ratio 2	0.73	0.18	0.04	0.03
Ratio 3	2.30	4.65	4.53	3.71
Total sugar (wt%)	45.57 ± 1.81 ^a^	41.45 ± 2.34 ^b^	30.87 ± 2.96 ^c^	40.29 ± 0.80 ^b^
Uronic acid (wt%)	31.87 ± 1.20 ^d^	41.56 ± 1.33 ^c^	58.54 ± 4.41 ^a^	52.83 ± 1.61 ^b^
Total phenolic (wt%)	0.30 ± 0.00 ^c^	1.36 ± 0.03 ^a^	1.28 ± 0.09 ^b^	0.18 ± 0.06 ^d^
Protein (wt%)	4.86 ± 0.19 ^b^	4.27 ± 0.89 ^b^	12.71 ± 1.40 ^a^	4.91 ± 0.57 ^b^
Mw (kDa)	4289.47	676.57	231.32	643.21
Methyl esterification degree (DM) (%)	19.34 ± 3.04 ^d^	72.07 ± 3.86 ^a^	32.11 ± 1.71 ^c^	38.67 ± 2.75 ^b^

Values followed by the different letters in the same column are significantly different (*p* < 0.05). -: not detected. Values for Rha, Ara, Gal, Glu, Xyl, and GalA are in relative mol%. Ratio 1 = GalA/(Rha + Ara + Gal). Ratio 2 = Rha/GalA. Ratio 3 = (Ara + Gal)/Rha. Ratios 1, 2, and 3 were calculated based on the mol % of monosaccharides.

**Table 2:** The chemical composition of pectins after reduction.

Chemical Features	CP	CP1	CP2	OP	OP1	OP2
Rha (%)	7.05 ± 1.67 ^c^	7.17 ± 0.34 ^c^	5.02 ± 2.40 ^c^	20.89 ± 0.71 ^a^	16.01 ± 3.61 ^b^	21.22 ± 0.83 ^a^
Ara (%)	12.91 ± 1.57 ^a^	8.00 ± 1.05 ^b^	8.99 ± 1.82 ^b^	3.03 ± 0.66 ^c^	3.22 ± 1.67 ^c^	2.08 ± 0.90 ^c^
Gal (%)	19.90 ± 2.71 ^d^	18.76 ± 0.46 ^d^	24.27 ± 0.45 ^c^	45.01 ± 5.79 ^b^	51.11 ± 1.35 ^a^	48.16 ± 1.63 ^bc^
Glu (%)	13.25 ± 1.74 ^a^	4.20 ± 1.39 ^b^	3.97 ± 0.63 ^b^	2.42 ± 1.30 ^bc^	1.86 ± 1.77 ^c^	1.18 ± 1.05 ^c^
Xyl (%)	6.76 ± 1.18 ^a^	1.10 ± 0.70 ^b^	1.15 ± 0.27 ^b^	-	-	-
GalA (%)	40.13 ± 6.51 ^b^	60.77 ± 2.34 ^a^	56.59 ± 0.49 ^a^	28.66 ± 4.63 ^c^	27.81 ± 2.41 ^c^	27.34 ± 2.65 ^c^
% HG = (GalA − Rha)	33.08	53.60	51.57	7.77	7.11	6.12
% RG-I = (2 Rha + Ara + Gal)	46.92	41.10	43.31	89.81	92.89	92.70
Ratio 1	1.01	1.79	1.48	0.42	0.33	0.38
Ratio 2	0.18	0.12	0.09	0.73	0.71	0.78
Ratio 3	4.65	3.73	6.62	2.30	3.24	2.37
Total sugar (wt%)	41.45 ± 2.34 ^b^	43.84 ± 0.67 ^bc^	43.27 ± 1.12 ^bc^	45.57 ± 1.81 ^a^	41.37 ± 1.16 ^b^	43.05 ± 0.89 ^bc^
Uronic acid (wt%)	41.56 ± 1.33 ^c^	64.37 ± 2.12 ^a^	59.64 ± 1.66 ^b^	31.87 ± 1.20 ^d^	28.23 ± 0.41 ^e^	31.47 ± 1.99 ^d^
Total phenolic (wt%)	1.36 ± 0.03 ^a^	0.33 ± 0.02 ^b^	0.29 ± 0.01 ^c^	0.30 ± 0.00 ^c^	0.34 ± 0.01 ^b^	0.05 ± 0.00 ^d^
Protein (wt%)	4.27 ± 0.89 ^a^	1.23 ± 0.31 ^bc^	0.94 ± 0.02 ^bc^	4.86 ± 0.19 ^a^	1.53 ± 0.26 ^b^	0.73 ± 0.20 ^c^
Mw (kDa)	676.57	65.68	18.18	4289.47	1620.59	119.12
Methyl esterification degree (DM) (%)	72.07 ± 3.86 ^a^	55.04 ± 2.92 ^b^	55.48 ± 1.23 ^b^	19.34 ± 3.04 ^d^	20.44 ± 1.31 ^d^	24.60 ± 1.37 ^c^

Values followed by the different letters in the same column are significantly different (*p* < 0.05). -: not detected. Values for Rha, Ara, Gal, Glu, Xyl, and GalA are in relative mol%. HG/% = galacturonic acid–rhamnose. RG-I/% = 2 rhamnose + galactose + arabinose. Ratio 1 = GalA/(Rha + Ara + Gal). Ratio 2 = Rha/GalA. Ratio 3 = (Ara + Gal)/Rha. Ratios 1, 2, and 3 were calculated based on the mol % of monosaccharides.

**Table 3:** Methylation analysis results of pectins: sugar residues and molar ratios.

Linkage Patterns	Mol Ratios (%)					
	CP	CP1	CP2	OP	OP1	OP2
T-Gal*p*	11.40	9.16	9.35	27.45	24.76	26.18
4-Gal*p*(A)	61.24	64.33	56.81	29.87	29.56	33.65
3,4-Gal*p*(A)	4.90	4.66	5.21	2.87	2.50	2.98
4,6-Gal*p*(A)	3.35	2.75	2.97	-	-	-
**Total galactose/galacturonic acid**	**80.88**	**80.90**	**74.34**	**60.19**	**56.82**	**62.81**
T-Ara*f*	2.02	2.76	4.89	-	-	-
2-Ara*f*	1.16	0.76	1.66	-	-	-
5-Ara*f*	0.91	1.01	5.95	-	-	-
2,3,4-Ara*p*	-	-	-	3.25	3.35	4.17
**Total arabinose**	**4.09**	**4.52**	**12.50**	**3.25**	**3.35**	**4.17**
T-Rha*p*	-	-	5.97	-	-	-
2-Rha*p*	2.32	3.78	2.47	6.95	8.47	4.30
2,4-Rha*p*	2.14	2.13	-	21.55	20.00	19.74
2,3,4-Rha*p*	-	-	-	2.45	1.64	2.11
**Total rhamnose**	**4.46**	**5.91**	**8.44**	**30.94**	**30.12**	**26.15**
T-Glc*p*	1.20	0.66	0.82	2.84	4.86	2.42
4-Glc*p*	9.38	7.31	2.91	2.78	4.85	3.23
**Total glucose**	**10.58**	**7.97**	**3.72**	**5.61**	**9.71**	**5.65**
T-Xyl*p*	-	0.70	0.99	-	-	-
**Total xylose**	**-**	**0.70**	**0.99**	**-**	**-**	**-**

Mol (%): The molar ratio of each sugar residue was determined by calculating the percentage of its peak area. -: not detected.

## Data Availability

The original contributions presented in this study are included in the article/Supplementary Materials. Further inquiries can be directed to the corresponding author.

## Supplementary Materials

The following supporting information can be downloaded at: https://www.mdpi.com/article/10.3390/nu17101738/s1, Table S1: List of forward and reverse primers used in this study; Table S2: Lifespan of *Drosophila* with different concentrations of pectins; Table S3: Lifespan of *Drosophila* with different molecular weights of pectins.

## Abbreviations

The following abbreviations are used in this manuscript:

HG	homogalacturonan
RG-I	rhamnogalacturonan I
RG-II	rhamnogalacturonan II
PPs	pectic polysaccharides
UC	ulcerative colitis
DSS	dextran sodium sulfate
GalA	Galacturonic acid
IBD	Inflammatory bowel disease
Rha	rhamnose
Ara	arabinose
Xyl	xylose
Gal	galactose
Glc	glucose
OP	okra pectic polysaccharide
CP	citrus pectic polysaccharide
AP	apple pectic polysaccharide
HP	hawthorn pectic polysaccharide
HPSEC	high-performance size exclusion chromatography
RID	refractive index detector
HPAEC-PAD	high-performance anion-exchange chromatography with pulsed amperometric detection
FT-IR	Fourier transform infrared spectroscopy
GC-MS	gas chromatography mass spectrometry
TFA	trifluoroacetic acid
PMAA	partially methylated alditol acetates
H&E	hematoxylin and eosin
DM	degree of methyl esterification
