# Supplementary material for: The Protective Effects of Pectic Polysaccharides on Dextran Sulfate Sodium-Induced Colitis in Drosophila melanogaster and Their Structure–Function Relationships
Source: Nutrients. 2025 May 20;17(10):1738. doi: 10.3390/nu17101738 (PMC12113875; doi:10.3390/nu17101738)
Supplement: Supplementary file 1 [file nutrients-17-01738-s001.zip › nutrients-3618317-supplementary.pdf]

Table S1. List of forward and reverse primers used in this study

Genes	Forward	Reverse
upd1	TCGCAACTTCGACGAGAACA	ATTGAAGCTCTGCCTGGGAC
upd2	AAGTTCCGCAAGGATCAGCA	GCTGGCGTGTGAAAGTTGAG
upd3	CAACTTCCGGCTGACCTTCC	GGCTTCAGATTGCAGGTGTTCT
Stat92E	AGCTGCAATCCTCCAGTTGTTC	CACCTCGTACAGCTCCACCTT
Sod1	TCAACATCACCGACTCCAAGA	CCCGTTGACTTGCTCAGCTC
Sod2	CGCCCTGGAGCCTATCATCT	GCTTGGTGGTGTCGCTCTTC
Cat	ACAAGGTCAAGAAGCGCACTC	CCAGACGCCATCCTCAGTGTA
Rp49	CACTTCATCCGCCACCAGTC	CGCTTGTTCGATCCGTAACC

Table S2. Lifespan of *Drosophila* with different concentrations of pectins

Sample	Concentration （mg/ml)	Average lifespan (h)	Median lifespan (h)	Maximum lifespan (h)
DSS		111.12 ± 10.09^{c}	112.80 ± 6.57^{a}	170.40 ± 10.04^{c}
	0.5	130.32 ± 11.53^{abc}	127.20 ± 13.68^{a}	192.00 ± 12.00^{abc}
OP	1	123.00 ± 8.50^{abc}	117.60 ± 15.65^{a}	189.60 ± 31.06^{abc}
	2	130.80 ± 11.50^{abc}	136.80 ± 10.73^{a}	187.20 ± 18.20^{abc}
	0.5	124.32 ± 16.55^{abc}	122.4 ± 29.88^{a}	206.40 ± 10.04^{a}
CP	1	122.40 ± 7.19^{abc}	120.00 ± 8.49^{a}	187.20 ± 21.80^{abc}
	2	137.28 ± 6.20^{ab}	141.60 ± 13.15^{a}	194.40 ± 17.80^{abc}
	0.5	119.28 ± 7.51^{abc}	124.80 ± 10.73^{a}	199.20 ± 13.68^{abc}
AP	1	120.96 ± 7.68^{abc}	120.00 ± 12.00^{a}	184.80 ± 16.10^{abc}
	2	126.00 ± 10.45^{abc}	129.60 ± 10.04^{a}	199.20 ± 16.10^{abc}
	0.5	117.48 ± 6.57^{bc}	122.40 ± 23.08^{a}	175.20 ± 6.57^{bc}
HP	1	115.92 ± 12.00^{c}	117.60 ± 19.72^{a}	177.60 ± 21.47^{abc}
	2	125.88 ± 10.95^{abc}	132.00 ± 12.00^{a}	187.20 ± 10.73^{abc}

Values followed by the different letters in the same column are significantly different ($p < 0.05$).

Table S3. Lifespan of *Drosophila* with different molecular weights of pectins

Sample	Average lifespan (h)	Median lifespan (h)	Maximum lifespan (h)	Average lifespan extension rate	Median lifespan extension rate	Maximum lifespan extension rate
DSS	104.88 ± 11.89^{c}	105.60 ± 15.65^{d}	172.80 ± 13.68^{c}			
CP	130.20 ± 10.03^{ab}	129.60 ± 10.04^{abc}	201.60 ± 15.65^{ab}	24.14%	22.73%	16.67%
CP1	127.32 ± 13.51^{ab}	124.80 ± 13.68^{bc}	199.20 ± 16.10^{b}	21.40%	18.18%	15.28%
CP2	143.85 ± 10.15^{a}	144.00 ± 9.80^{a}	219.00 ± 11.49^{a}	37.16%	36.36%	26.74%
OP	128.40 ± 12.51^{ab}	127.20 ± 13.68^{abc}	192.00 ± 12.00^{b}	22.43%	20.45%	11.11%
OP1	119.88 ± 15.36^{cd}	115.20 ± 13.68^{cd}	189.60 ± 10.04^{bc}	14.30%	9.09%	9.72%
OP2	134.60 ± 5.57^{ab}	136.00 ± 13.86^{ab}	196.00 ± 6.93^{b}	28.34%	28.79%	13.43%

Values followed by the different letters in the same column are significantly different ($p < 0.05$).
